# Rewiring Drug Research and Development through Human Data-Driven Discovery (HD^3^)

**DOI:** 10.3390/pharmaceutics15061673

**Published:** 2023-06-07

**Authors:** David B. Jackson, Rebecca Racz, Sarah Kim, Stephan Brock, Keith Burkhart

**Affiliations:** 1 Molecular Health GmbH, 69115 Heidelberg, Germany; stephan.brock@molecularhealth.com; 2 Division of Applied Regulatory Science, Office of Clinical Pharmacology, Center for Drug Evaluation and Research, US Food and Drug Administration, Silver Spring, MD 20993, USA; rebecca.racz@fda.hhs.gov (R.R.); keith.burkhart@fda.hhs.gov (K.B.); 3 Department of Pharmaceutics, Center for Pharmacometrics and Systems Pharmacology, College of Pharmacy, University of Florida, Orlando, FL 32827, USA; sarahkim@cop.ufl.edu

**Keywords:** systems pharmacology, polypharmacology, adverse events, drug discovery, functional genomics, disease modeling, network analysis, innovation

## Abstract

In an era of unparalleled technical advancement, the pharmaceutical industry is struggling to transform data into increased research and development efficiency, and, as a corollary, new drugs for patients. Here, we briefly review some of the commonly discussed issues around this counterintuitive innovation crisis. Looking at both industry- and science-related factors, we posit that traditional preclinical research is front-loading the development pipeline with data and drug candidates that are unlikely to succeed in patients. Applying a first principles analysis, we highlight the critical culprits and provide suggestions as to how these issues can be rectified through the pursuit of a Human Data-driven Discovery (HD^3^) paradigm. Consistent with other examples of disruptive innovation, we propose that new levels of success are not dependent on new inventions, but rather on the strategic integration of existing data and technology assets. In support of these suggestions, we highlight the power of HD^3^, through recently published proof-of-concept applications in the areas of drug safety analysis and prediction, drug repositioning, the rational design of combination therapies and the global response to the COVID-19 pandemic. We conclude that innovators must play a key role in expediting the path to a largely human-focused, systems-based approach to drug discovery and research.

## 1. Introduction

The past three decades have provided some of the greatest technological advancements in human history. Never before have we had such a wealth of data and knowledge about living systems. The sequencing of the human genome, our ability to routinely probe the diverse molecular composition of living cells, the practical realization of Moore’s Law and the rapid evolution of artificial intelligence, are but a few of the many advances that helped spawn a new era of hope for the betterment of human health. When viewed against the background of an ever-aging society and debilitating global pandemics of infectious agents and chronic disease, such hope is more than warranted. However, an unbiased appraisal of modern drug discovery suggests that a commensurate impact on drug development success is still wanting [1]. Indeed, empirical evidence demonstrates that drug development success rates have decreased over time, while attrition rates, development times, and required investment have all increased considerably [2]. On the surface, it looks as though most companies are finding it difficult to transform these technological advancements into improvements in research and development (R&D) efficiency and, as a corollary, new and effective medicines. As this trend continues, critical questions for the industry and patients remain: why is this the case, and how might we rectify it?

## 2. Eroom’s Law and the Innovation Crisis

Several authors have sought to dissect the roots of this apparent “innovation crisis”, quantifying both the costs of developing new drugs and the diverse factors impinging on success rates; two critical elements in the overall efficiency of the R&D process. While there is limited transparency regarding the cost of innovation in the pharmaceutical industry, a variety of studies have been reported. Using only publicly available data, Wouters et al., analyzed the costs associated with the drugs approved by the US Food and Drug Administration between 2009 and 2018 [3]. Out of 355 potential candidates, financial details were available for a total of 63 drugs (18%), coming from 47 mostly small to mid-sized companies. Median capitalized R&D investment was estimated at USD 985.3 million, and a mean investment of USD 1335.9 million was estimated as required to bring a new drug to market. Median estimates also varied according to the therapeutic area, ranging from USD 765.9 million for neurology drugs to USD 2771.6 million for oncological and immunomodulating agents.

Complementary studies by DiMasi et al. used confidential data for 106 products developed by 10 large pharma companies to propose a base case mean cost estimate of around USD 2800 million [4]. These higher estimates potentially reflect a combination of higher clinical costs incurred by larger developers and lower estimates of trial success for each stage of development. Despite their differences, such studies emphasize the sheer scale of investment required to bring a new drug to market. In fact, no other industry invests so much in product development as the pharmaceutical industry. This is perhaps not surprising, given the trajectory of market growth, with global prescription drug sales increasing from USD 649 to USD 768 billion between 2008 and 2016, with estimates prior to the COVID-19 pandemic predicting USD 1060 billion in sales by 2022 [5].

Given such enormous financial commitment and opportunity, one might naturally expect parallel improvements in the overall rate of innovation and R&D efficiency—as measured by the number of newly approved drugs divided by the total overall costs of development. Unfortunately, however, empirical evidence again suggests that we have witnessed a declining rate in overall R&D efficiency over the past number of decades. Scannell et al. reported that the all-in cost of R&D on new drugs approved by the US FDA has risen exponentially for 60 years [6], leading to an associated exponential decrease in the overall rate of R&D efficiency. More specifically, the authors showed that the number of new FDA approved drugs per billion US dollars of R&D spend was halved approximately every 9 years between 1950 and 2010. They characterized this phenomenon as ‘Eroom’s Law’ i.e., the inverse of Moore’s Law. The potential causes for Eroom’s Law have been widely discussed in the literature, but before we examine the details it is instructive to first review the high-level details of the current drug R&D paradigm.

### 2.1. The Traditional Drug Discovery Paradigm

The drug discovery process typically begins with the search for target molecules that are either directly or indirectly associated with a disease of interest [7]. Once identified, this target must be validated by demonstrating a potential therapeutic effect through modulation [8]. Then, an assay is developed and screening is performed, in search of one or more lead compounds that can modulate its function in a therapeutically desirable manner, often with the additional help of structure-based drug design and virtual screening [9]. These lead compounds undergo preclinical evaluation in in vitro and/or in vivo model systems, to characterize potential pharmacological and toxicological behaviors. After further lead optimization through medicinal or computational chemistry [10], a single compound is usually selected, patent applications are filed and an Investigational New Drug Application (IND) is submitted to regulators. If successful, the sponsor can then proceed to the first of three phases of clinical testing in humans.

Development begins in phase Ia, where a single dose is administered to a group of healthy volunteers, followed by phase Ib, where increasing doses are used to assess safety, pharmacokinetic, and pharmacodynamic parameters. In an optimal case, additional studies are performed at this stage to decipher and/or confirm the precise mechanism of action. If no critical safety issues are identified, the compound may move to phase II, where it is tested directly in several hundred patients in an attempt to glean further insights into its clinical efficacy, adverse event profile and optimal dosing regimen. What ultimately emerges is a clearer picture of the risk–benefit profile. In the final phase III of the study, the effectiveness of the compound is examined, normally in a randomized control trial (RCT) setting with thousands of patients, where its effectiveness is compared to either a placebo or a current standard of care. Once successfully completed, the sponsor submits a New Drug Application (NDA) to the regulatory authorities, and if successful the drug can be launched on the market. Thereafter, further Phase IV clinical trials can be used to demonstrate the real-world safety and effectiveness of the drug or to compare it to other treatment modalities, in so-called pragmatic clinical trials.

The current drug R&D paradigm can therefore be viewed as a set of contiguous steps, where the financial outlays are compounded at each stage, leading many to adopt the “fail early and fail fast” approach. One can also view the process as a hypothesis filtration funnel, where new innovations have effectively increased the width of the funnel by, for example, increasing the efficiency of target identification, e.g., through application of functional genomic methodologies, such as RNAi and the CRISPR-cas9 system [11]. Through the widening of the funnel, the cumulative effect of all these advances should, in theory, lead to the negation of Eroom’s Law, but compelling evidence of this is still wanting. The issues involved can be divided into two general categories of challenge, (a) industry-related challenges, pertaining to the nature and regulation of the pharmaceutical industry, and (b) scientific challenges, pertaining to prevailing discovery modalities and accepted research norms. In the following section we review some of these issues, and then hone in on what we believe to be the most fundamental, first-principle causes.

### 2.2. Industry-Level Challenges

Industry-related challenges are often discussed, although in our opinion they are probably not the primary drivers of the innovation crisis. In this section, we introduce three of the most commonly quoted factors.

#### 2.2.1. Regulatory Oversight

Post-marketing safety issues surrounding drugs such as Vioxx [12] and Avandia [13] have encouraged greater regulatory stringency in the pre- and post-approval assessment of drugs. In response to such problems, the FDA issued the Amendments Act, which enabled the agency to stipulate risk evaluations prior to approval and additional clinical studies when post-marketing safety problems emerge [14]. While regulatory stringency is sometimes quoted as a challenge, it is clear that the remit of agencies is to ensure an appropriate risk:benefit ratio for each treatment and target population. As such, the process can be viewed as an essential element of the drug development process and is unlikely to play a significant role in perpetuating the innovation crisis. On the contrary, high safety standards can only be good for patients. There is also evidence that higher standards raise incentives to develop more innovative drugs, and are generally associated with higher international market success [15].

#### 2.2.2. The Drug “Innovation Chasm”

Another issue that has certainly impacted the overall return on investment is the number of “me-too” and “follow-on” drugs. Developing a drug for an indication where an effective therapy already exists means that companies must demonstrate that the new drug has credible advantages over the approved standard. This has been referred to as the “Better than the Beatles” problem [6], but one can also think of it as an ever-expanding “innovation chasm”, where with every newly approved drug in a specific indication it becomes more and more difficult for newer drugs to cross the chasm to broader market acceptance. To do so often requires larger investment, for example, due to larger and/or longer clinical trials. The Tufts Center for the Study of Drug Development (CSDD), for example, looked at 377 drugs and biologics approved by the FDA between 2008 and 2018 and found that while an average of 83.1 clinical trial months was required between 2008 and 2013, this number rose to 89.8 months in the following 5 years (2014–2018) [16]. With increasing time comes greater financial outlay, thus exacerbating the R&D efficiency challenge.

#### 2.2.3. Mergers and Acquisitions

High R&D costs, expiring patents and impending generic competition, have also forced larger companies to pursue a mergers and acquisitions strategy. Many empirical studies suggest that M&As can have negative effects on the innovative freedoms of the companies involved [17]. Financial pressures can force the closure of entire R&D projects, or even entire research sites, especially those of the acquired party. This may be particularly damaging if companies use M&A to escape innovation competition by buying others with related competing pipelines and terminating their development. Furthermore, important intangible resources may be irreparably damaged through loss of ‘knowledge capital’ with the exit of key scientists. Thus, by restricting the R&D freedoms of smaller, more innovative firms, there is evidence that M&A activity can compound the innovation crisis.

### 2.3. Science and Technology Challenges

Preclinical drug discovery is both dichotomous and reductionist in nature. The overall process was transformed in the 1990s, when the strategy of low-throughput in vivo physiological screening and medicinal chemistry optimization (i.e., phenotype-based drug discovery) [18] became largely supplanted by the target-focused, high-throughput screening (HTS) of large compound libraries (i.e., target-based drug discovery) [19]. Several radical innovations in molecular and computational technologies have recently transformed the scale and specificity of both approaches. Advances in the automation and parallelization of in vitro and in vivo models, and improvements in imaging technologies and artificial intelligence (AI)-driven image analysis, have all contributed to the re-emergence of phenotype-based screening as an effective discovery strategy. The target-based approach has also been revolutionized through advances in genomics, transcriptomics, proteomics, transgenic/humanized animal models and CRISPR/RNAi libraries [20]. Combinatorial chemistry, which led to a proliferation in the size and composition of chemical libraries for target-focused HTS, has also been complemented by the advent of structure-based drug design and related approaches such as fragment-based drug design [21]. Taken together, these advances should have revolutionized the return on investment in drug discovery research. However, while transforming it quantitatively speaking, important qualitative issues prevail, many of which are likely debilitating.

#### 2.3.1. Target-Based Discovery

**Figure 1:**
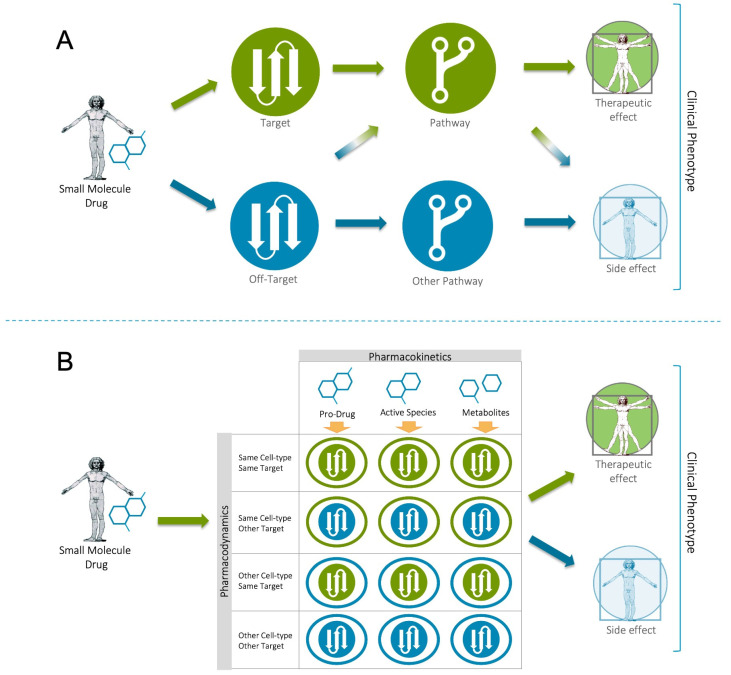
(**A**)**.** Schematic overview of drug mode of action (MOA) according to the Target-Based Discovery paradigm. (**B**). Schematic overview of drug MOA according to the HD^3^ paradigm. Here, single drugs and their metabolites can interact with target proteins in the target cell/tissue, or in other tissues. In addition, the drug can interact with different targets in the target tissue and/or in other tissues. This complexity highlights the need for cell-type specific-drug MOA models.

While the single-target focus holds many practical efficiencies, it is clearly of greater relevance to the ‘one gene–one disease’ theory, including both inherited diseases (e.g., the delta508Phe deletion in CFTR associated with cystic fibrosis) [22] and certain cancer driver mutations (e.g., BCR-ABL in chronic myeloid leukemia (CML)) [23]. Today, fewer than 700 human proteins have been established as targets of approved drugs, most of which belong to either the kinase, GPCR or ion channel protein families [24]. Given that this represents less than 1% of the human proteome, the “low-hanging fruit” hypothesis has been proposed, suggesting that most of the obvious discoveries and easy-to-validate targets have already been found, leaving only more complex biomedical challenges for the industry to solve. This might certainly be true for complex neurological disorders such as Alzheimer’s or metabolic syndrome. One of the most fundamental problems with the single-target strategy is that we know that most diseases are multifactorial in nature and that single targets can play different functional roles across different cellular systems (see Figure 1). Targets exist in highly structured and tightly integrated cellular information transduction systems that extend from nucleic acids to proteins to diverse biochemical molecules (e.g., lipids, carbohydrates, metabolites). Moreover, complex disease etiology likely arises because both protein function and expression are controlled by the quaternary structure and/or network context in which the protein exists.

#### 2.3.2. Drug Promiscuity

We also know that small-molecule drugs, such as protein kinase inhibitors, tend to act at the level of several targets, and that such molecular promiscuity (also termed polypharmacology) is critical to their clinical effects. To estimate the scale of this phenomenon, Mestres et al. analyzed various drug:target databases to estimate the average number of targets per drug, identifying a total of 1.8 for the DrugBank database of curated therapeutic targets, and 2.7 for the WOMBAT database of drug:target affinities. When both validated targets and affinity-based targets were considered, the number increased to an average of 6.3 targets per drug [25]. Thus, in the absence of broader knowledge about the systemic interactions of targets and small molecules, it is unsurprising that many promising paths of therapeutic development eventually fall victim to the poorly predictable intricacies of human molecular diversity. Moreover, such knowledge is key to understanding the entire clinical-utility and risk profile of all small-molecule drugs.

#### 2.3.3. The Reproducibility Crisis

Another critical issue relates to the robustness and reproducibility of preclinical results. It is often assumed that the peer-review process ensures that only scientifically valid results are published, and that they are described in enough detail to allow reproduction in other laboratories. Drug discovery is dependent on accurate and relevant results, as these form the basis of prudent decision-making along the discovery and development value chain. However, just as our ability to generate massive tomes of new data has increased exponentially, so too have concerns about the reliability of most preclinical findings. Empirical studies have estimated that 75–90% of preclinical results from high-profile journals are not reproducible in independent experiments [26,27,28]. These findings are not specific to in vitro studies. For example, Hackham et al. reported [29] that only about a third of highly cited animal model research actually translated into randomized human trials. In some specific therapeutic areas, such as strokes, no evidence of translatability to patients has been found, despite massive investment [30].

#### 2.3.4. The Problem with Model Systems

Congruence between in vitro and animal models and the corresponding human disease is still accepted as a fundamental tenet of biomedical research. Yet this is an assumption that receives relatively little objective analysis at the research strategy level. Much pre-clinical pharmaceutical research is still today strongly dependent on target-focused in vitro assays and animal models of human disease. While this provides us with a wealth of new data and peer-reviewed insights, the question remains as to whether this new information is in any way representative of the human disease state. Patient cells do not normally grow on plastic, there is no circulation in vitro, nutrient/oxygen supplies are different and the cellular microenvironments are completely artificial (e.g., presence of antibiotics, different endocrine concentrations). In fact, the list of differences between cell culture and in vivo systems is so extensive that it warrants broader objective analysis and critique. Animal models, too, clearly have significant problems. For example, it is commonly observed that the disruption of a gene in one genetic background of a mouse might cause severe phenotypes, such as lethality, whilst in another strain it might be phenotypically innocuous. If such discrepancies can occur at the level of a single model organism, how can they reliably translate to the development of drugs for human disease?

To better understand this issue, it is instructive to consider the distinction between the “structural biochemistry” of the cell and its “functional biochemistry” [31]. Structurally speaking, we can reasonably assume that if a particular biochemical reaction is possible in vitro, then it is also likely to be possible in vivo. By examining such biochemical events in vitro, it is possible to construct a network of connected biochemical events in vivo. However, an in vitro system can tell us little about the intricacies of the functional biochemistry within different cellular systems. Given that it is the functional biochemistry that is perturbed in most human diseases, all model systems hold critical weaknesses in the study of human disease. While such fundamental problems appear to be largely ignored, due to underlying practicalities, they are likely having negative impacts on the overall return of investment. This begs the important question: are we relying on false principles, when we should instead be turning to the first principles of human biology?

## 3. The “First Principles” Case for a Human Data-Driven Discovery (HD^3^) Paradigm

The aforementioned issues, although incomplete, provide a good indication of the scale and diversity of the innovation challenge. However, in our opinion, it is the scientific issues that deserve immediate analysis and introspection. To do this, we can borrow from one of the numerous innovation frameworks that have successfully been applied to solve complex social, engineering and technology challenges. These include design thinking, computational thinking, analytical thinking, lateral thinking and first principles thinking. Arguably, the most successful is first principles thinking (FPT), an approach first practiced by Aristotle, who defined it as the search for “the fundamental basis from which a thing is known” [32]. In practice, innovators are encouraged to critically question every assumption of the challenge and break it down into basic components, and to search for solutions from the “first principles”. From this, new learnings and opportunities are defined. This approach is distinct from reasoning by analogy (e.g., using competitive analysis), which builds knowledge and solves problems based on prior assumptions, beliefs and the widely held ‘best practices’ approved by the majority of people.

Applying FPT to the drug discovery process, we have seen that much of preclinical research is built upon the inherent practicalities of the model systems, as opposed to the functional realities of human biology. At the data level, such an approach has quantitative advantages, but not qualitative. Therefore, failure to expeditiously balance investment towards human-specific approaches risks us applying new technologies to generate even more incongruent data. As a corollary, we can posit that current standards of preclinical research are front-loading the drug discovery process with data that lacks human specificity and technical reproducibility for routinely developing successful new drugs. This is ultimately what may be responsible for long development times and high attrition rates. Thus, pivoting discovery to a largely human-focused approach using human-specific data and in silico disease models (i.e., Human Data-Driven Discovery: HD^3^), has the potential to provide radical improvements in drug research efficiency.

FPT analysis also reminds us that while the target-based discovery paradigm provides a useful simplification, it is only rarely consistent with biological reality. When administered to a patient, drugs interact with a plethora of molecular entities across different cellular systems, a profile we refer to as the drug “biotype”. Understanding the relationship between a drug biotype, its chemical features (here termed “chemotype”) and its resulting clinical effects (i.e., phenotype) is essential to understanding the molecular basis of the drug mode of action. FPT helps us to reduce the challenge to an even more simplistic fact. Fundamentally, it is the three-dimensional arrangement of atoms in a small-molecule drug (i.e., the chemotype) that interacts with the complimentary binding pockets in targets throughout several cellular systems (i.e., the drug biotype) to induce network-level changes that result in emergent clinical effects (i.e., the phenotype). Instead of thinking of the lock-and-key analogy for the drug and target, the system-based perspective is more akin to that of a combination lock, or a “dial on a safe”, where only certain chemotypes possess ‘the code’ (i.e., biotype) to induce specific phenotypic effects. This simple perspective also emphasizes the key weakness of model systems. Structural- and network-based differences in target paralogues within the model mean they are unlikely to induce the same phenotypic response to a chemotype as observed in a human—the model system biotype is simply too different. With this simple perspective in mind, we now examine some of the factors that can help us transition to a more “humanized” drug discovery paradigm, with the power and relevance of human data at the core of our focus.

### Human Data as a Driver for Systems-Based Discovery

Today, the molecular characterization of human populations plays a critical role in target-driven discovery. In oncology, for example, the application of omics technologies has helped characterize the key molecular differences between treatment responders and non-responders, thereby enabling the development of efficacious therapies targeting specific driver mutations, such as imatinib [33] and crizotinib [34]. However, one of the primary advantages of model systems is that they also allow us to prospectively perturb the function of genes in the study of disease-specific targets and to perform in vivo physiological screening with small molecule chemistries. While it is generally understood that such studies are unethical in patients, the reality is that doctors have for decades been performing physiological phenotypic studies on their patients with prescribed drugs. Once administered, the drug and/or its metabolites interact with targets in different cellular systems. The phenotypic readout is a consequence of the combined molecular interactions across the entire patient system (see Figure 1B). From this perspective, we can view a side effect as a drug-induced disease phenotype, whose molecular etiology tells us something not only about the drug’s mode of action, but also about the targets/pathways associated with the observed phenotype. Thus, if we can accurately define a drug’s interaction partners/biotype (targets, off-targets, metabolizing enzymes, transporters) across all cellular systems, we can learn more about the human-specific molecular networks involved in human phenotypes. The advantage of this approach, in comparison to model systems, is that the clinical observations and connected molecular knowledge are directly defined by the human condition.

**Figure 2:**
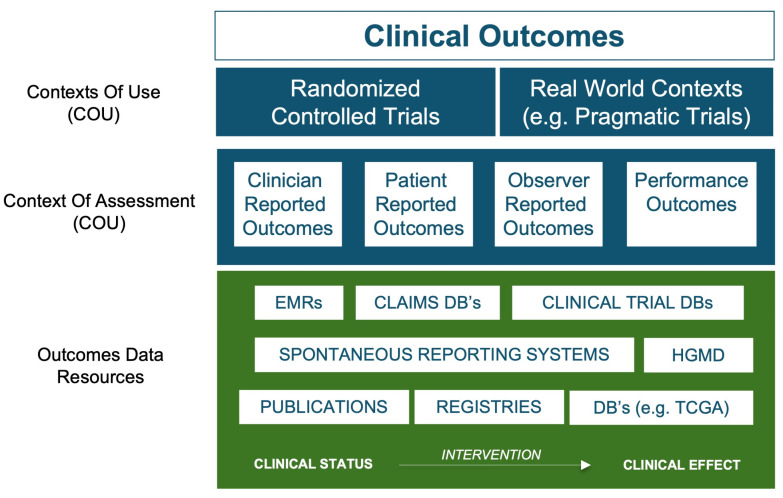
Overview of key sources of clinical outcome data. Beyond randomized controlled trial (RCT) results, there are four types of outcome reports, otherwise known as “contexts of assessment”, associated with RWD: (1) clinician-reported outcomes (e.g., from electronic medical records (EMRs)), (2) patient-reported outcomes, (3) observer-reported outcomes and (4) performance outcomes. While clinician-reported outcomes are the most reliable source of outcome data types, there is an ever-growing realization of the value of patient-reported outcomes. Nevertheless, for the purposes of the HD^3^ approach, it is treatment outcome data that provide the most valuable datapoints, with spontaneous reporting systems such as FAERS and Vigibase providing a highly accessible source of this information for tens of millions of patients. We can also extend the concept of “outcome” to include the phenotypic consequences of genetic aberrations, with such data being available in disease-agnostic databases such as the Human Gene Mutation Database (HGMD) and OMIM, or disease-specific databases such as The Cancer Genome Atlas project (TCGA).

An important facilitating factor in the pivot to a human-focused discovery paradigm is therefore the utility of the treatment and outcome information from the vast tomes of existing real-world data (RWD) and clinical trial results. RWD exist as a spectrum of different qualities, typically defined by both the context of the assessment from which the data were derived and the degree to which the data were generated, to answer specific research questions (see Figure 2). Data pertaining to treatments and clinical outcomes, whether positive (e.g., drug-induced disease remission) or negative (e.g., disease recurrence or adverse reactions) are of primary importance. Such data are widely available in research databases, large-scale clinical registries, EMR-linked sources, administration/claims sources, facilitated networks and regulatory databases. Spontaneous reporting system databases are particularly interesting, and although redacted in terms of clinical narratives, they offer a highly valuable window into observed drug-induced adverse event (AE) phenotypes for millions of patients. Major sources include the FDA’s Adverse Event Reporting System (FAERS) [35] and Sentinel Initiative [36,37], together with the European Medicines Agency EudraVigilance system [38] and the global database of individual case safety reports (ICSRs) called VigiBase, maintained by the World Health Organization’s (WHO) Uppsala Monitoring Center (UMC) [39]. The data contained in these databases are analogous to chemical phenotype screening data from model systems, only this time specific to humans. By providing insights into the phenotypic effects of drug-induced perturbation on targets within the patient system, they allow us to capitalize on publicly available treatment and outcome data for tens of millions of patients.

Treatment outcome data can also be integrated with data emerging from the multi-omics characterization of human populations. A vast wealth of data resources and public genomics data initiatives have become available over the years, with general, organ-specific and disease-specific data now globally accessible (for a list of 86 different globally available resources, see Supplementary Table S1). Here, disease-associated genetic data from resources such as the Online Mendelian Inheritance in Man (OMIM) database [40] or the phenotype-associated NHGRI-EBI Genome-Wide Association Study (GWAS) catalog [41] are of particular interest. Updated daily, the OMIM database catalogues information for more than 15,000 genes with a core emphasis on the molecular relationship between genetic and phenotypic diversity, especially around human disorders. The data also lends itself to organ-specific analyses (and modeling), with Parsa et al., for example, compiling a list of the 258 OMIM genes responsible for kidney-related diseases, including renal hypoplasia, dysplasia or agenesis, end-stage renal disease and proteinuria [42]. By aligning this data with GWAS data from the CKDGen Consortium, they were further able to characterize the potential association of genetic polymorphisms and kidney function within the general population [43]. Although not reported by the authors, such data can also be further contextualized with kidney-specific disease pathway information, such as the Kidney and Urinary Pathway Knowledge Base (KUPKB) (http://www.kupkb.org/, accessed on 18 January 2023) [44] or the Chronic Kidney Disease database (CKDdb) (http://www.padb.org/ckddb, accessed on 18 January 2023) [45], or with data from drugs used to treat kidney diseases and outcome data related to kidney-specific side-effects from other drug treatments.

The power of such strategies was also demonstrated by results from the TCGA project, where molecular data and phenotypic information have been analyzed to decipher novel targets and prognostic classifiers. Analysis of the TCGA endometrial carcinoma dataset, for example, has brought important new insights into the molecular nature of this disease, including the discovery of a new classification system based on four prognostically significant subgroups [46]. Indeed, it can be argued that most recent clinical advancements emerge from analysis of patient-derived molecular data. However, vast tomes of clinical information available throughout the healthcare system remain underutilized for discovery purposes.

Two other types of data resource are fundamental to systems-based discovery, (a) network models of disease systems and signaling pathways, defined initially at the level of proteins (nodes) and their interactions (edges), and (b) accurate and comprehensive drug-to-target knowledge. The network view provides a basic proteo-anatomical structure of the system, upon which additional molecular data sources can be added. The scale-free and redundant characteristics of these networks often permit perturbation without a complete loss of function, implying that multiple perturbations, at nodes and/or edges, are typically involved in the emergence of disease phenotypes. Recent computational work by Zhong et al., for example, examined the system-level mutational features of heritable disease, and found that they were more likely to be caused by mutations at edges, as opposed to nodes [47]. Interestingly, edge-based perturbations, typically involving in-frame mutations of the (near) full-length protein, were more commonly observed across multiple diseases. Such mutations tend to abrogate interaction with one or more neighboring nodes. Moreover, different disease phenotypes may be caused by different mutations in a single edge. Nodal mutations, on the other hand, typically involved truncated proteins and did not necessarily affect the interaction with other proteins nodes in a signaling network. Drug-to-target knowledge is also a key prerequisite in systems-based discovery endeavors: oi addition to the aforementioned DrugBank [48] and WOMBAT [49] databases, the Therapeutic Targets Database (TTD) [50], Pubchem [51] and ChEBI [52], all provide critical knowledge for systems-based analyses of drug targets.

This brings us to the next level of the challenge. How do we optimally structure this drug and network knowledge to facilitate drug discovery? Ontologies will certainly play a critical role in making data not just machine readable, but also machine actionable. A key element here will be ontology interoperability and robust ontology applications to help make data Findable, Accessible, Interoperable and Reusable (i.e., FAIR compliant) [53]. Beyond the ontological challenges associated with knowledge modeling, the biological accuracy of systems-based models is critical, especially if we are aiming for whole-patient models. Today, most pathway models represent an amalgamation of biochemical findings across a multitude of different cellular systems, under various physiological conditions. This leads to generic model representations that are likely inaccurate at the cell-type-specific level. We must therefore aim for cell-type and tissue-specific representations of core biological mechanisms, which can then be mapped at the whole-patient level. Several existing resources can aid this endeavor, including important work by Jiang et al., who reported a quantitative proteome map of the human body, with expression data for 12,000 genes across 32 normal human tissues [54]. Other examples include The Human Protein Atlas (HPA), which presents the spatial distribution of proteins in 44 different human tissues and 20 cancer types [55]. Organ-specific sources are also widely available, such as The Brain Protein Atlas [56] and the Human Kidney and Urine Proteome Project (HKUPP) (http://www.hkupp.org/, accessed on 23 January 2023) [57]. The resultant networks facilitate the mapping of drugs to phenotypes across all levels of the patient system and provide a powerful basis for hypothesis generation and AI-driven discovery. They also allow us to add additional data types (e.g., genomic, transcriptomic, drug-binding constants and target-activity data) that may later facilitate more functional analyses using, for example, simulation algorithms. Such an approach would largely meet the requirements for developing an effective in silico model system, since the human disease/systems and the in silico models should be substantially congruent with respect to structure and composition.

Finally, from a technical perspective, outcome data such as spontaneous AE reports are typically stored in relational databases (RDBs), in multiple tables with information pertaining to the case demography, drugs (medications) given, reported AEs, etc. Such data structure allows the easy integration of new reports, distribution and sharing of data and a relatively straightforward retrieval of specific/individual information. However, the need to join a large number of tables to combine information for each outcome, AE, medication and co-medication, indication and demography, can result in rather complex queries and long computational times. Moreover, a thorough understanding of the underlying data structure is required to avoid potential pitfalls, such as the multi-axiality of data, leading to erroneous results that are not always directly apparent. These characteristics can make advanced analyses of data in the RDB structure more cumbersome and impractical. One of practical solutions is to convert the RDB into a graph database for the purpose of data analysis. Graph databases (GDBs) store information in the form of nodes (entities) and their properties as edges (relationships), instead of tables with rows and columns [58]. As each node is directly connected to all the other relevant nodes, the queries to retrieve more ‘distant’ information are much simpler and faster, as there is no need to join multiple tables. More importantly, the GDB structure enables the use of efficient algorithms from simple random walks to graph convolutional networks, to facilitate the discovery of hidden relationships between entities and the preparation/transformation of data for machine learning/AI approaches, such as feature engineering. Several reports show that the use of GDBs in the analysis of safety signals or predicting the safety of drugs show that such approaches have the potential to outperform the current approaches [59,60,61,62,63]. The future development of knowledge graphs integrating full outcomes and spontaneous AE report databases, together with other information, will not only improve the performance of drug toxicity predictions but also help to uncover hitherto unknown interactions between drugs and co-morbidities. From this perspective, the approach may prove quite effective in target deconvolution and drug (re-)positioning studies.

## 4. Current Applications of the HD^3^ Approach

### 4.1. Application to the Analysis and Prediction of Adverse Events

Some of the most successful proof-of-concept applications of HD^3^ systems-based discovery have come from the analysis and prediction of adverse event data. This is likely driven by the public availability of AE data for millions of patients, with Vigibase alone containing over 30 million case reports. The data itself is de-identified, and typically comprised of basic patient information (e.g., gender, weight and age), the drugs they were prescribed, the adverse events that were observed, and the associated clinical outcomes. Traditional analysis of this data by pharmacovigilance (PV) teams uses disproportionality statistics such as proportional reporting ratios (PRRs), to quantify the relative congruence of the binary drug with the AE relationships. Bayesian correlation models and a variety of machine learning algorithms have also successfully been applied, but all such approaches lack molecular insight. From a more conceptual standpoint, we can view AE data as a form of human phenotypic screening data, where doctors administer drugs that impinge on the activity of several proteins within the patient system, eliciting AEs. Such AEs can be considered drug-induced diseases, whose molecular mechanisms tells us something about the underlying disease etiology. Given that such resources cover millions of patient lives, they are highly valuable resources for HD^3^-based approaches, such as those described next.

Work by Soldatos et al. demonstrated the feasibility of transforming 8.2 million FAERS case reports into as many patient-specific systems pharmacology models [64]. Given that drug names are reported without a controlled dictionary, the authors first implemented the mapping of reported drugs to unique chemical IDs, resulting in the unequivocal integration of about 95% of all FAERS reports. Drugbank was then used to link these drugs to their targets, with further levels of molecular integration achieved by mapping all target proteins to their respective pathways within the Reactome [65] and PID (NCI-Nature and BioCarta) [66] databases. The network was further extended through the integration of the ATC ontology [67] and text-mined literature pertaining to all binary relationships captured within the network. Using this process, patient case reports detailing drug-induced phenotypes were linked to knowledge from 9 entity types: 2600 drugs were linked to 1800 targets, 201 metabolizing enzymes, 103 transporter proteins, >1000 pathways, and 881 ATC drug classes, all of which were connected to clinical data pertaining to 15,400 indications, 19,300 adverse reactions and 7 clinical outcomes. As a result, circa 8 million systems-based patient models were generated that could be queried at the level of any entity and/or compared either directly or through user-defined patient cohorts. For example, instead of just comparing the AEs of drug A versus drug B, it is possible to query AEs from the perspective of target A versus target B, or pathway A versus pathway B. Beyond the analysis of potential off-target effects, the authors also demonstrated the utility of the platform (MH Effect) in predicting adverse events for developmental drugs, the rational design of combination therapies, and target-based drug interactions. Similar platforms have since emerged, including AbbVie’s Off-Target Safety Assessment (OTSA) technology [68] and Clarivates OFF-X system [69], with OFF-X and MH Effect being utilized by regulatory scientists.

Building on this general approach, Kim et al. recently reported two interesting proof-of-concept studies. Firstly, they explored the underlying molecular mechanisms causing trastuzumab-induced cardiotoxicity, and how the rate of toxicity may change due to drug–drug interactions at the molecular-pathway and target levels [70]. Trastuzumab is a targeted-therapy drug targeting HER2, which is overexpressed in 25% of all breast cancer patients. HER2 blockade can cause cardiotoxic side effects, because the HER2 receptors are present in not only breast cancer cells but also normal cardiomyocytes. Among the ~750 molecular mechanisms found by mapping the FAERS data to chemical and biological databanks, the mechanisms related to the apoptosis regulator proteins which are members of the BCL-2 family were found to have a statistically significant association with trastuzumab-induced cardiotoxicity, which aligns with other reports [71,72]. The researchers further explored how the concomitant use of other drugs affects the possible molecular mechanisms related to mitochondria dysfunction in cardiomyocytes. Doxorubicin, which is often given with trastuzumab, also has a high risk of cardiotoxicity. The PRRs between cardiotoxicity and both trastuzumab and doxorubicin were higher than 40. The combination cohort of these two drugs indicated a ~2.5-fold increase in the PRR. Other potential different pathways related to mitochondrial biogenesis and function (i.e., peroxisome proliferator-activated receptor-𝛾 coactivator 1-𝛼 (PGC-1𝛼 or PPAR𝛼) and PPARβ) were found, which might induce a synergistic effect leading to the increased risk of developing cardiotoxicity. In addition to the combination of trastuzumab and doxorubicin, drug–drug interactions that mitigate trastuzumab-induced cardiotoxicity were also explored for the concomitant use of other drugs, i.e., tamoxifen, paroxetine, and lapatinib, with trastuzumab. For the mechanisms decreasing the toxicity, the highlighted molecular mechanisms were the anti-apoptotic effect through calcineurin-dependent pathways, the antioxidant activity of glutathione, and the adenosine monophosphate-activated protein kinase activation.

Secondly, the approach was also applied to untangle the complexity of the underlying molecular pathways and targets of adverse events of immune checkpoint inhibitors [73]. Their study focused on how mechanistic differences between cytotoxic T-lymphocyte antigen-4 (CTLA-4) and programmed death-1 (PD-1) affect colitis, by mapping the FAERS data to the associated molecular pathway and target levels. The PRR indicating the statistical association between a drug and colitis was ~3 times higher for ipilimumab (the anti-CTLA-4 drug), compared to the PRRs for nivolumab and pembrolizumab (anti-PD-1 drugs). They hypothesized that the severer toxicity of the anti-CTLA drug would be because of its early responses in the sequence of boosting T-cell activation. While there were limited data to drive statistically significant evidence with respect to the mechanistic causality, the researchers’ work elucidated how to apply the reverse translational systems-based approach to predict the drug safety profile.

#### Examples from the FDA’s Division of Applied Regulatory Science

Clinical Pharmacologists at the FDA’s Division of Applied Regulatory Science (Center for Drug Evaluation and Research (CDER)) are often tasked with examining potential molecular mechanisms to support or negate emergent safety signals from pharmacovigilance (PV) endeavors. In contrast to the traditional PV, these so-called “biological plausibility consultations” utilize a palette of bioinformatics and chemoinformatics applications to examine both target- and systems-based mechanisms. Target-focused assessment has been termed “target adverse event” (TAE) analysis, to emphasize that real-world safety data is being integrated and analyzed at the level of the target/system, as opposed to the traditional drug-based view. The combined output of PV and TAE analyses is then used to determine whether enough mechanistic evidence exists to support a label change for marketed drugs.

In one example, researchers at CDER analyzed the biological plausibility of montelukast, by inducing neuropsychiatric events such as hallucinations, suicidal thoughts, depression, and sleep disturbances. Target analyses for neuropsychiatric events found drugs that bind serotonin, dopamine, and acetylcholine muscarinic receptors or act on protein targets that increase or decrease neurotransmission for these neurotransmitters to be highly associated with neuropsychiatric events. Montelukast binds to the serotonin receptor 5-HT2B, dopamine receptor 3, muscarinic receptors 1 and 3, and dopamine and norepinephrine reuptake transporters. Structurally, montelukast has a quinoline moiety that is found in other drugs that cause neuropsychiatric events (see the tafenoquine label [74]). This analysis provided biological plausibility for the FDA’s decision to add neuropsychiatric events to the montelukast label [75].

TAE and systems-based analyses have provided signal strengthening to support safety label changes for other drugs. In evaluating a signal of seizures and gadolinium contrast agents, infusion reactions or anaphylactoid reactions were also noted. Histamine release enhances cholinergic neurotransmission, which can include seizures. Further analyses noted that other drugs associated with infusion reactions also had reports for seizures in FAERS and the literature. Monoclonal antibodies that bind to the proprotein convertase subtilisin kexin type 9 (PCSK9) were associated with flu-like illness. Analyses found a disproportional number of flu-like symptoms for this class of drugs and other monoclonal antibodies that bind cytokines, but not for the statin drugs that are taken concomitantly. Finally, as pimavanserin is a highly specific 5-HT2a receptor antagonist, this target and other comparator drugs supported the addition of ‘falls’ to the label.

Multiple case reports also suggested a relationship between second-generation antipsychotics, such as risperidone and olanzapine, and serotonin syndrome. Therefore, FAERS was utilized in combination with molecular target information within the MH Effect system to develop a hypothesis of the mechanism for serotonin syndrome. Based on this combined data set, 5-HT2A antagonism and 5-HT1A agonism were identified as common mechanisms for second-generation antipsychotic-associated serotonin syndrome. Additionally, FAERS and several case reports supported that interactions between second-generation antipsychotics and other serotonergic agents may increase the risk of serotonin syndrome. These hypotheses were further supported by a literature search. Therefore, this study demonstrated that computational analyses of FAERS data using molecular knowledge can easily generate hypotheses for adverse event mechanisms [76].

While improving the overall evidence-based use for drug re-labelling decisions, the above examples are still retrospective in nature. The FDA has, however, also expressed a strong interest in developing more proactive methods to predict AEs before they happen. Such a capability can assist both in the post-market surveillance of AEs and in the risk assessment during and after IND application. In recently reported proof-of-concept work, scientists used TAE profiles for six test drugs to aggregate data from FAERS and FDA drug labels, based on drug targets. The goal of this study was to predict the evolution of the drug’s label for four years post approval. A genetic algorithm was utilized to set thresholds for features in these datasets to maximize performance. Utilizing on safety data available prior to the approval of each drug, the method correctly identified 78% of postmarket label changes, and had a precision of 67%, a recall of 81%, and a specificity of 71% [77].

In follow-on work, the authors attempted to improve the performance of the previous adverse event prediction model by utilizing target-adverse event profiles; additional drugs, learning features, and algorithms were evaluated [78]. In addition to the FAERS data and drug labels, the new model incorporated data from EMBASE, a biomedical literature database, as a feature. A larger set of 55 drugs were utilized in the study, and predictions were made for 36 groups of adverse events. Finally, an ensemble model containing Naïve Bayes, K-Nearest Neighbor, Support Vector Machine, and C4.5 was applied to make predictions. Utilizing this updated model, overall precision improved to 72%, recall decreased to 70%, and specificity increased to 86%, although these metrics can be tuned to a user’s specifications. Additionally, 69% of label changes were correctly identified. The results of these studies demonstrated promise for the target-adverse event profile approach of predicting adverse events.

Concurrently with the development of the ensemble model, a Naïve Bayes approach was utilized to predict 135 individual adverse events for 54 drugs. Similar to the ensemble model study, adverse event information from FDA product labels and the scientific literature was utilized, and additional features such as structural and target similarities and duration of post-market experience were incorporated. The evaluation was performed using a probabilistic approach of over 10,000 iterations: 53 of the 135 events demonstrated a high probability of having a high positive predictive value, and many of these events had well-characterized target–event relationships. Additionally, 32% of the predicted drug label changes occurred. The ensemble model and the Naïve Bayes model have been used concurrently to identify potential safety events for new drugs [79].

### 4.2. Application of HD^3^ to Drug Repositioning and Combinatorial Therapy Design

Polypharmacology seeks to capitalize on systems-level knowledge about disease etiology to define either a single drug that binds two or more targets or two (or more) drugs that bind to two (or more) targets. The mechanistic goal of these strategies is to recalibrate the perturbed network processes underlying the disease state. Polypharmacology is not only the key to exploiting off-target effects in drug repurposing endeavors, but also to addressing more complex disease etiologies and adaptive resistance mechanisms. With the molecular analysis of patient tumors revealing extraordinary levels of genetic complexity and clonal adaptability, oncology has provided an optimal testing bed for the development of these strategies. To redress such a challenge, we require systems-level knowledge as to how multiple nodes in a signaling network combine to produce the pathophysiology of disease. Such models, while typically “proteo-centric”, also include disease-specific molecular perturbations, such as copy number variations, point mutations and differential expression. Combining empirical molecular knowledge through a clinically focused network perspective allows us to identify combinatorial opportunities in a physiologically relevant manner. Beyond drug discovery, the strategy also holds particular promise for personalized oncology, with the mapping of molecular data from a patient’s tumor to system-specific models, allowing the identification of personalized drug combinations.

The targeted design of combination therapies provides an interesting strategy for drug repositioning. This was elegantly demonstrated by recent discoveries around beta adrenergic receptor (BAR) inhibition, via the beta-blocker class of drugs. The first hints of the role of BAR in cancer came from studies indicating that psychosocial factors such as stress, depression, and lack of community might promote tumor growth and progression. In ovarian cancer in particular, such effects were found to directly enhance tumor pathogenesis by protecting tumor cells from anoikis, promoting tumor cell invasion and tumor-associated angiogenesis. The molecular mechanism was found to be mediated via tumor cell ADRB2 (an adrenergic receptor), with phospho-proteomic analysis demonstrating that ADRB signaling leads to Src activation via a unique PKA-mediated mechanism [80]. This network was found to be critical to the regulation of phospho-proteomic signaling associated with ovarian cancer progression. Importantly, these observations were combined with real-world data from FAERS, to examine the death rate of patients treated with ADRB2 inhibitors, compared to the death rate in a cohort without. Here, “death rate” was used as a surrogate marker for treatment efficacy, with a lower death rate implying greater efficacy. Analysis revealed that mortality was reduced across major cancer types in patients where ADRB2 signaling was inhibited with beta-blockers, which, together with the lab findings, identified BAR inhibition as a potential combinatorial route to anti-cancer treatment.

Several clinical studies have now provided evidence for repositioning beta-blockers, either alone or in combination with approved cancer therapies. For example, a phase-2 pilot study (NCT01265576) examined ADRB2 inhibition in hepatocellular carcinoma (HCC) patients, where sorafenib was combined with the beta-blocker propranolol and a COX2 inhibitor. The combination was found to increase therapy duration and overall survival, compared to sorafenib alone [81]. Propranolol is a nonselective beta-blocker that was first approved in 1967 for cardiovascular indications. Since then, it has been repositioned with respect to a wide range of indications, including the essential tremor and prophylaxis of migraines, and as a first-line treatment for problematic infantile hemangiomas [82]. More recently, Fjaestad et al. reported that co-administration of propranolol significantly enhanced the efficacy of anti-CTLA4 therapy [83], whilst Amaya et al. found that beta-blockers increased progression-free and overall survival in patients diagnosed with metastatic angiosarcoma [84].

Several other promising examples of positioning non-oncology drugs in the treatment of cancer have been reported, including itraconazole, statins [85], metformin, aspirin, digoxin, and pantoprazole. Itraconazole is a potent anti-fungal drug that inhibits lanosterol-14α-demethylase in the cell membrane of the fungus [86]. Interestingly, it also shows anticancer activity across a number of solid and hematological cancer types. While the mechanism remains unclear, evidence points to the modulation of several components of the signaling pathways involved in apoptosis and cell cycle arrest, including AMPK, mTOR, Wnt/β-catenin and Hedgehog [87]. Interestingly, Cheng et al. used complex network theory to develop three supervised inference methods for drug repositioning, namely target-based similarity inference (TBSI), drug-based similarity inference (DBSI) and network-based inference (NBI) [88]. With its superior performance, NBI predicted potent polypharmacological effects of itraconazole, ketoconazole montelukast, diclofenac, and simvastatin on estrogen receptors and the dipeptidyl peptidase-IV enzyme, which were later validated in in vitro binding assays. Related studies showed that itraconazole synergizes with paclitaxel in the inhibition of endothelial cells, raising hopes for its use in combination therapy for endothelial cancer [89].

In other work, Huang et al. used multiomic patient datasets and the Broad Institute’s Connectivity Map (CMap) database to identify members of the cardiac glycoside family, including digoxin, as potential treatments for medulloblastomas [90]. CMap (available as CLUE for commercial users) is a cloud-based data analysis platform for perturbational datasets generated using gene expression (L1000) and proteomic (P100 and GCP) assays. Several different forms of in silico analyses have also pointed to the potential clinical utility of metformin in cancer. Clinical meta-analyses and in vitro studies have together pointed to metformin as a potential treatment option in several solid tumor types, including breast, pancreas and lung. Sun et al. [91], developed a metformin specific signaling pathway network (SPNetwork) using their Drug-specific Signaling Pathway Network resource (DSPathNet). Using gene enrichment analyses from type 2 diabetes and cancer GWAS studies, network analyses and literature mining, the authors identified seven enriched genes (PPARGC1A, CDKN1A, MYC, ESR1, MAX, STK11, and SP1) impinging on a novel Myc-associated pathway that they proposed played a key role in metformin’s anti-cancer MOA.

Beyond oncology, HD^3^ also played a critical role in the global response to the recent COVID-19 pandemic. As highlighted by Sheridan et al., numerous large-scale data and AI initiatives were launched to provide a testbed for pandemic forecasting and response [92]. At the time, knowledge connecting intricate clinical manifestations of COVID-19 with molecular underpinnings was fragmented and new findings were being reported daily, thus exacerbating the challenge to stay abreast of emergent knowledge. In response, several groups developed COVID-19 knowledge models. For example. Domingo-Fernández et al. used knowledge graphs (KGs) to develop an integrated COVID-19 model. KGs have several advantages for systems modeling, as they provide a means to capture, represent and formalize structured information. KGs are also complemented by a broad range of algorithms that partially automate the process of knowledge discovery. Their open-source model could be used as a framework for target identification and drug repositioning efforts [93].

**Figure 3:**
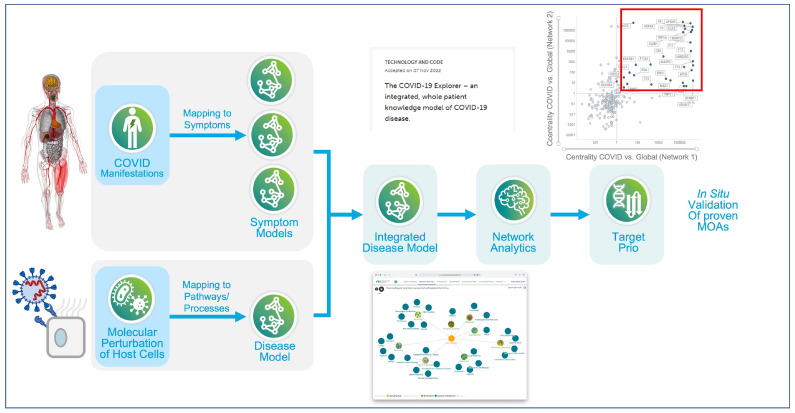
Schematic of the knowledge modeling process used by Brock et al. to produce a whole-patient knowledge model of COVID-19 symptomatology. The process used a previously reported SARS-CoV protein interaction map1 of 332 high confidence interactors as a seed for knowledge expansion. This was achieved through inclusion of factors from the host cell, host-specific response (e.g., innate immune response) and associated phenotypes. Next, this “seed model” was further expanded to include protein interactors, associated pathways and regulatory information. This “base model” was further expanded through integration of key molecular protagonists associated with COVID-19 pathophysiology and symptomatology including common disease symptoms (e.g., anosmia), severe manifestations (e.g., acute respiratory distress syndrome (ARDS)), outcome- and severity-associated risk factors (e.g., age) and diseases with COVID-19-like symptoms. Symptomatology associated ‘pre-models’ were then manually curated. Linkage of these pre-models through related molecular protagonists resulted in a comprehensive and fully interactive whole-patient COVID-19 disease model representation, providing a link between key molecular disease mechanisms and eight core pathogenic processes: inflammatory signaling, coagulation, barrier permeability, senescence, autoimmunity, fibrogenic signaling, nociception and exocytosis. Finally, these mechanisms were linked with the respective symptoms and associated pathogenic pathways and affected organ systems. Results were then made available via the open source COVID-19 explorer (https://covid19.molecularhealth.com, accessed on 8 January 2023) and used as a basis for drug repurposing endeavors.

In more recent work, researchers from the ETH in Zurich reported the generation of a whole-patient knowledge model of COVID-19 symptomatology. The authors used the commercially developed Dataome technology to extract and combine emergent clinical and molecular data around COVID-19 [94]. A total of 332 high-confidence virus–host interactors from SARS-CoV-1 were used as a seed for knowledge expansion. Next, this “seed model” was further expanded to include protein interactors, associated pathways and regulatory information. The resultant “base model” centered on a converging molecular phenotype that included the host proteins responsible for virus entry, TMPRSS2 and ACE2, together with significantly differentially down-regulated components of the interferon-stimulated genes (ISG) induced by the virus infection (ACE2 and SERPING1). This base model was then further expanded through the integration of genes associated with COVID-19 pathophysiology and symptomatology, including a) common disease symptoms (e.g., fatigue), b) severe manifestations (e.g., acute respiratory distress syndrome (ARDS)), c) outcome- and severity-associated risk factors (e.g., age) and d) diseases with COVID-19-like symptoms. Symptomatology associated ‘pre-models’ were then structured by domain experts, and then linked through related molecular protagonists (see Figure 3).

The resultant whole-patient model (available at http://covid19.molecularhealth.com, accessed on 8 January 2023) revealed that SARS-CoV-2 perturbs eight core pathogenic processes: inflammatory signaling, coagulation, barrier permeability, senescence, autoimmunity, fibrogenic signaling, nociception and exocytosis [95]. Together, these mechanisms were proposed to be responsible for unleashing a pathogenesis spectrum, ranging from ‘a perfect storm’ triggered by acute hyper-inflammation to chronic fatigue in protracted COVID-19 syndromes. Importantly, when viewed at the whole-patient level, it appears that the ever-growing list and complexity of seemingly unrelated clinical symptoms can be related to a limited set of molecular mechanisms. Although the model was initially based on data-mining hypotheses, many of the predicted clinical phenotypes (e.g., Kawasaki-like syndromes) and molecular mechanisms were subsequently reported in the peer-reviewed literature, thus providing both a real-time and real-world validation of their knowledge model. The work also presented several drug repositioning opportunities, including spironolactone, atorvastatin, and losartan, many of which have since entered clinical trial.

## 5. Synopsis and Future Directions

The emergence of Eroom’s Law and the associated innovation crisis has run counter to the levels of technical invention that have been achieved in recent decades. Reviewing the evidence, we posit that currently accepted norms in drug discovery are causing us to frontload the drug development pipeline with preclinical data that bears little congruence with the human in vivo condition. Here, we emphasize the need to “humanize” the entire drug discovery process, and propose Human Data-Driven Discovery (HD^3^) as a focal point for these endeavors. HD^3^ clearly provides a rational framework for structuring clinical and molecular evidence around drug polypharmacology and mode of action (MOA). By providing more physiologically representative in silico models of current knowledge, it provides a way to improve the accuracy of both expert-driven hypothesis generation and data-/AI-driven discovery. Today, this in silico model-based framework is already augmenting a variety of existing research avenues, including the rational design of combination therapies, drug (re-) positioning, and the analysis and prediction of adverse events. Indeed, with the COVID-19 pandemic providing excellent validation of the power of human-focused systems-based discovery, compelling evidence that HD^3^ approaches might improve the quality of candidates entering the development pipeline is starting to emerge.

In an update to their initial description of Eroom’s Law, Ringel et al. recently revisited the issue to examine the trajectory of R&D efficiency over the last decade [96]. Their results provide at least initial validation that HD^3^-based approaches may now be starting to positively impact drug R&D efficiency. By 2018, the downward trendline in R&D efficiency, as measured by the number of new molecular entities (NMEs) approved per USD billion spent, appeared to have broken out to the upside, with an additional 0.7 NMEs approved between 2010 and 2018. Two major factors seem to be driving this change; (a) the availability of better information for decision making, and (b) the improved utility of that information. Importantly, they hypothesize that better information appears to derive from increasing focus on targets that have been validated using human-derived data (e.g., from GWAS studies). This position is supported by other recent studies suggesting the positive impact of human data on drug R&D efficiency [97]. A common theme appears to be that data quality (i.e., human-derived data) is a superior success factor to data quantity (e.g., high-throughput data from model systems). Interestingly, Astra Zeneca also recently reported the strategic 5R framework (Right Target, Right Tissue, Right Safety, Right Patient, Right Commercial), that helped transform their R&D productivity and supported a return to business growth. [98]. The central focus of the 5R initiative was a move to increased utility of “humanized models”, such as patient-derived xenograft models, organs-on-a-chip technology, humanized miniature organs, and 3D bioprinting. Interestingly, the strategy also appears to have had important intangible effects on personal biases, with researchers tending to adopt a more “truth-seeking” behavior, in contrast to the over-optimism or fear of failure that so often accompanies research endeavors. Their transformative success is further evidence that the innovation crisis is best tackled by efforts to “humanize” our current drug discovery paradigm, a premise also supported by the many proof-of-concept examples presented in this review.

We have provided a very simple first principles perspective on why we must expeditiously pivot to a primarily HD^3^ drug research paradigm. At the most fundamental level, it is the three-dimensional arrangement of atoms in a small-molecule drug (i.e., the chemotype) that interact with the typically numerous proteins in biological systems (i.e., the biotype) to induce network-based changes that ultimately result in clinical effects (i.e., the phenotype). Defining the relationship between these three core components not only provides a focus for re-wiring the utility of current data and technology assets, but is also a stark reminder of the shortcomings of non-human model systems. Differences in structural complementarity between a human drug and the paralogous ligand binding domains within model systems likely become amplified at the whole-system level, which can seriously obfuscate the relevance and utility of any phenotypic observation or result. Even small structural changes in the complementarity between drug and paralogous target can have profound functional consequences. Thus, while model systems are extremely helpful in deciphering the structural biochemistry of the cell, from a functional perspective, they are simply too far removed from the in vivo realities of human disease. Notwithstanding, before we can consider an in silico whole-patient systems medicine approach, we must first focus on getting the simplest elements of the modeling process right, particularly the comprehensive drug:target knowledge and system-specific disease models.

**Figure 4:**
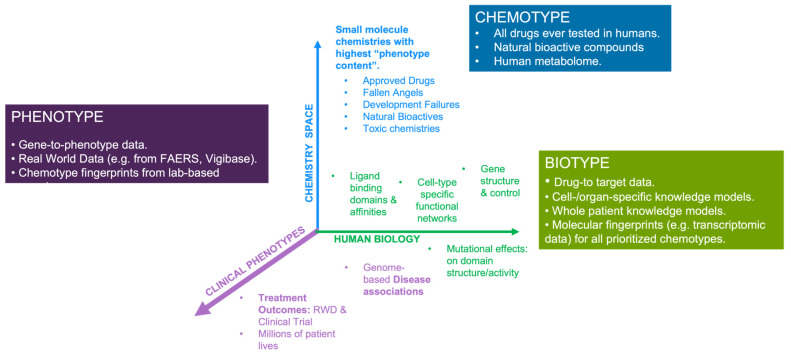
Schematic overview of the components and priorities associated with drug chemotypes, biotypes and associated phenotypes. We believe that this simple categorization can facilitate the structuring of efforts around the HD^3^ approach. While key datapoints are highlighted in the graph, data priorities are provided in the boxes.

We described several recent examples of the HD^3^ approach in action, both at the level of drug discovery and at the level of applied regulatory science, which emphasize the power of deciphering the relationship between a drug chemotype and biotype and the resultant clinical phenotypes. We believe the tripartite nature of the challenge (chemotype → biotype → phenotype), provides a useful framework for a technical focus. For example, with respect to chemotypes, we know that the available chemical space is astronomical in dimension, with estimates ranging from 10^26^ and 10^62^ [99] chemotypes that would comply with the Lipinski guidelines for oral drugs. Borrowing from James Flood’s famous quote that “the best way to discover a new drug is to study an old one”, a rational starting point for rewiring the drug discovery process is to focus modeling efforts on all chemicals ever tested in humans. While we could not find any peer-reviewed estimate of precise numbers, they are likely to be in the tens of thousands. Often associated with phenotype data (e.g., side effect information), these chemicals represent the most feature-/knowledge-rich part of the chemical space, and thus lend themselves to the power of AI-based discovery methodologies. Later, other important components, such as the human metabolome and bioactive chemicals from natural food sources will also be important in extending the power of the approach. For all components of this “human chemome”, broader efforts are required to capture the full extent of their biotypes, i.e., their polypharmacology and molecular paths across different cellular systems within the patient. This requires a focus on experimental and curation efforts towards defining accurate and comprehensive target information for each drug. Publicly available drug-to-target knowledge resources still have significant weaknesses, including, (a) limited target coverage, (b) false positives (e.g., genes regulated by the drug), (c) metabolizing enzymes and transporters classified as targets, and (d) limited information on the effect of mutations on target binding affinities. It also requires a clear focus on the generation of cell-type-specific knowledge models that accurately capture the structural biochemistry of cellular systems across all organs. As emphasized by the COVID-19 knowledge modeling work, individual cell and tissue models can later be combined at the level of whole-patient knowledge models—enabling us to optimally link drug treatments with observed clinical symptomatology and outcomes. A summary of the associated data priorities for these endeavors is provided in Figure 4.

A key shortcoming of traditional approaches to disease modeling is the assumption that cellular organization occurs primarily at the proteomic level, with proteins often acting sequentially, to provide a structural framework for a cellular response to diverse environmental cues. While this convenient simplification allows us to build useful models of protein pathways, it obviates a critical question. Where does the specificity of pathway/protein complex membership come from? This cannot be explained by complementarity alone at the level of protein:protein interaction domains. With many proteins possessing the same interaction domain, how is it that the right protein partner is found within the vast expanse of the cellular milieu? The answer no doubt lies within poorly appreciated genomic signatures that regulate the co-transcription and co-translation of otherwise distant genes coding for protein members of the same pathway/quaternary structure. Here, features such as gene promoters and enhancers will likely play a role, but the fact remains that such “signatures” have yet to be properly characterized or integrated within cellular models. From an in silico modeling perspective, this argues for the need to move beyond “proteo-centric” models of human disease, to include signatures at the level of diverse biological features, from genomic to proteomic to cellular to human-phenotype levels.

Outcome data from clinical trial and real-world contexts is of prime importance to the HD^3^ paradigm. Beyond the vast availability of disease- and organ-specific multi-omics data, we have also highlighted how treatment outcome data can provide important insights into a drug’s molecular mode of action. By matching a patient’s prescribed drugs to their associated targets, we can transform raw clinical data into in silico patient models [63]. The targets thus provide the biological integration point for the systems-level analyses of observed clinical outcomes, further emphasizing the importance of comprehensive drug-to-target knowledge. Beyond the plethora of RWD sources mentioned, outcome data is also available from commercial entities such as IQVIA, and other governmental agencies such as the Center for Medicare/Medicade Services (CMS), which alone hold data for hundreds of millions of treated patients, covering thousands of drugs and diseases. Importantly, as patient empowerment continues to grow, so too does the availability of patient reported outcomes, with social media platforms, digital therapeutics, and online forums such as PatientsLikeMe providing a wealth of potential insight. Physiological parameters are also being captured through wearable mhealth solutions, and are bringing us closer to an era where every patient will be defined and analyzed in the context of their own big data—our so-called digital twin. In this context, we can envisage the advent of whole-patient disease models as not only a framework for AI-driven drug discovery, but also for their utility in precision medicine, where a systems-based analysis of patient data can be used to prescribe or contraindicate treatments.

**Figure 5:**
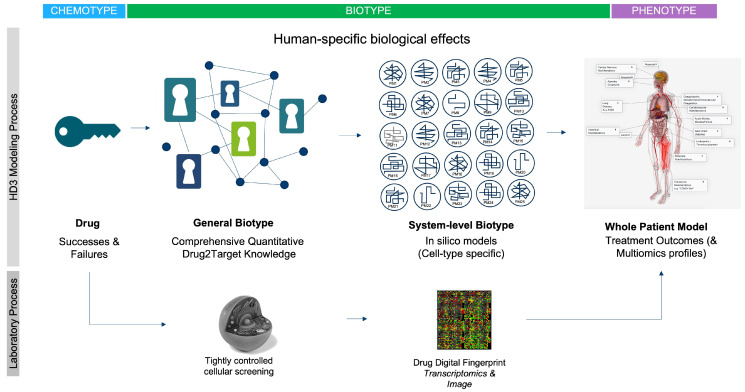
High-level schematic showing how HD3 systems-based discovery can be integrated into tightly controlled drug fingerprinting assays to evolve whole-patient molecular models for hundreds of millions of individual patients. Key to this approach is prioritized drug space, complete drug-to-target knowledge, and network models (i.e., cell-type or tissue/organ specific), integrated onto whole-patient knowledge frameworks. Using this approach, new uses for old drugs may be found, the area of failed drug space may again be explored, and novel chemistries can be analyzed.

It is important to emphasize that it is not our intention to negate the critical role that in vitro/in vivo model system research has played in drug discovery, but rather to reiterate the important existing and too-often ignored weaknesses. The human body is a universe of molecular complexity, and although HD^3^ data can provide only a snapshot, the information content is still directly congruent with the goal of conquering human disease. Notwithstanding, model systems will likely continue to play an important role in drug discovery, for example in the study of infectious disease and toxicology, but their general relevance will likely become increasingly dependent on the strategic alignment with the principles of the HD^3^ approach. For example, focusing on the aforementioned “human chemome”, we could use cell-based screening to evolve drug-specific molecular fingerprints of drug action; a “molecular phenotype” of sorts (see Figure 5). The goal of these fingerprints is not to help us define the drug’s MOA, but rather the pattern of molecular effects within a standardized biological system. In an integrated strategy, well-controlled and replicated transcriptomics studies could be used to produce such fingerprints, which would then be mapped to the associated whole-patient knowledge models for each drug. The same fingerprinting process can be applied to new molecular entities, which can then be analyzed and interpreted against the combined HD^3^ knowledge framework. Similar strategies are being applied by innovative new AI-driven drug discovery companies that have evolved over the past five years (for a list of 230 AI-driven drug discovery start-ups, see [100]). Using their current pipelines as a surrogate for R&D success, these innovators provide the hope that “humanizing” the drug research and discovery paradigm can indeed expedite the path to important new therapies for patients. We can further hope that their success might expedite the transformation of the entire biopharmaceutical industry to a primarily human-focused discovery paradigm.

## 6. Conclusions

Borrowing from learnings across other industries, we now know that most disruptive innovations do not necessarily derive from new inventions, but rather from the strategic integration of existing data and technology assets. Embracing this reality, we believe that all the right elements are now in place to expeditiously pivot our drug discovery endeavors towards a primarily Human Data-Driven Discovery (HD^3^) paradigm. Advances in omics technologies, computing, and AI analytics can now interface with enormous tomes of human multi-omics datasets and under-utilized sources of treatment outcome data for hundreds of millions of patients, to make this a viable proposition today. Combined with more comprehensive definitions of drug polypharmacology, and the structuring of data within cell-/tissue-/organ-specific in silico models, the HD^3^ strategy will allow us to evolve whole-patient models of disease symptomatology and drug MOA, initially for hundreds of millions of patients. These can serve as the perfect knowledge framework for technologies such as GraphDB and the many exciting machine learning methodologies that continue to evolve apace. This can also eventually provide the basis for more accurate dynamic simulations of drug activity and disease mechanisms. Emergent evidence from early adopters validates the effectiveness of the HD^3^ approach, particularly in the areas of drug safety analysis/prediction and drug (re-)positioning. In contexts where it has been applied, intangible benefits have also been observed, such as encouraging “truth seeking” behavior amongst scientists, as opposed to over-optimism and fear of failure. Nevertheless, much remains to be done to close the gaps in even the simplest of challenges, including the generation of comprehensive drug:target knowledge, starting with all approved drugs and the generation of accurate systems-specific disease models. Finally, it may ultimately serve the ethically important goal of decreasing our reliance on animal model research. From these perspectives, the HD^3^ paradigm stands to not only “humanize” drug discovery, but also to make it more humane. This is the kind of disruptive innovation that is required to provide the maximum socio-economic impact and expedite the path to safer and more effective therapeutics for all patients.

## Data Availability

Not applicable.

## Supplementary Materials

The following supporting information can be downloaded at https://www.mdpi.com/article/10.3390/pharmaceutics15061673/s1, Table S1: 86 Human Genomics Data Initiatives from around the world.
